# Quercetin Induces Apoptosis in HepG2 Cells *via* Directly Interacting with YY1 to Disrupt YY1-p53 Interaction

**DOI:** 10.3390/metabo13020229

**Published:** 2023-02-03

**Authors:** Hui Guan, Wenyuan Zhang, Hui Liu, Yang Jiang, Feng Li, Maoyu Wu, Geoffrey I. N. Waterhouse, Dongxiao Sun-Waterhouse, Dapeng Li

**Affiliations:** 1 Key Laboratory of Food Processing Technology and Quality Control of Shandong Higher Education Institutes, College of Food Science and Engineering, Shandong Agricultural University, 61 Dai Zong Street, Tai’an 271018, China; 2 Jinan Fruit Research Institute of All China Federation of Supply & Marketing Cooperatives, 16001 East Road Jingshi, Jinan 250220, China; 3 School of Chemical Sciences, The University of Auckland, Auckland 0632, New Zealand

**Keywords:** quercetin-YY1 interaction, anticancer, apoptosis, YY1-p53 binding, binding competition

## Abstract

Quercetin is a flavonol found in edible plants and possesses a significant anticancer activity. This study explored the mechanism by which quercetin prevented liver cancer *via* inducing apoptosis in HepG2 cells. Quercetin induced cell proliferation and apoptosis through inhibiting YY1 and facilitating p53 expression and subsequently increasing the Bax/Bcl-2 ratio. The results revealed that YY1 knockdown promoted apoptosis, whilst YY1 overexpression suppressed apoptosis *via* direct physical interaction between YY1 and p53 to regulate the p53 signaling pathway. Molecular docking using native and mutant YY1 proteins showed that quercetin could interact directly with YY1, and the binding of quercetin to YY1 significantly decreased the docking energy of YY1 with p53 protein. The interactions between quercetin and YY1 protein included direct binding and non-bonded indirect interactions, as confirmed by cellular thermal shift assay, UV-Vis absorption spectroscopy, fluorescence spectroscopy and circular dichroism spectroscopy. It was likely that quercetin directly bound to YY1 protein to compete with p53 for the binding sites of YY1 to disrupt the YY1-p53 interaction, thereby promoting p53 activation. This study provides insights into the mechanism underlying quercetin’s anticancer action and supports the development of quercetin as an anticancer therapeutic agent.

## 1. Introduction

Cancer has risen in prominence as a primary cause of mortality due to the fast increase in, and age of, the world population [1]. Liver cancer is now the sixth most prevalent cancer worldwide, making it a major public health concern (https://www.wcrf.org/cancer-trends/liver-cancer-statistics, accessed on 1 December 2022). Natural therapeutics have been actively pursued to prevent and suppress various cancers, due to the toxicity and severe adverse effects induced by synthetic agents or chemical drugs and their absorption inhibition and/or resistance against other treatments. Among the natural therapeutics, flavonoids have attracted increasing attention, as they not only possess significant antioxidant, anti-inflammatory, anticarcinogenic and hepatoprotective effects [2], but also improve response and reverse resistance to other anticancer treatments [3]. Flavonoids have demonstrated anticancer properties against a range of cancers [4]. Quercetin, as a typical flavonol-type flavonoid, has been shown to exert significant inhibition against numerous different types of cancers, including liver, breast, pancreatic, lung, colon and prostate cancers and so on [5]. 

The mechanisms underlying flavonoids’ anticancer effects have been linked to their abilities to regulate reactive oxygen species (ROS) and the activities of antioxidant enzymes, modulate signal transduction pathways, promote cell cycle arrest, induce apoptosis, initiate autophagy, inhibit cancer cell proliferation and invasiveness, downregulate glycolytic metabolism, and lower the risk of metastasis [6,7,8]. The structural features of quercetin empower its potency in these aspects, including its B ring o-dihydroxyl groups, 4-oxo group-linked 2,3-alkene, and 3- and 5-hydroxyl groups. Quercetin has been shown to have anticancer properties in cell cultures, animal models and epidemiological studies [9,10,11]. In in vitro studies, quercetin was shown to induce apoptosis and reduce the proliferation and migration in multiple human cancer cell lines, including ovarian cancer cells, hepatocellular carcinoma (HCC) cell lines, prostate carcinoma cells, breast cancer cells and pancreatic cancer cells [11,12]. In a human hepatoma cell line (HepG2), quercetin could induce apoptosis *via* the inhibition of fatty acid synthase [13]. Interestingly, quercetin was found to induce cytotoxicity and death in carcinogenic cells (leukemic and breast cancer cells) through DNA fragmentation, cell cycle arrest and apoptosis activation, but the similar concentrations did not influence normal cells derived from human embryonic kidneys (293T) and mouse embryonic fibroblasts (MEF-1) [14]. In vivo studies, quercetin was also found capable of inducing apoptosis of cancer cells to suppress cancers. Intravenous administration of polymer micelle-encapsulated quercetin in mice led to a significant inhibition of xenograft A2780S ovarian tumor growth *via* initiating apoptosis of cancer cells and suppressing angiogenesis [11]. Quercetin pretreatment enhanced the anti-ovarian-cancer effect of cisplatin while protecting the kidneys against damage [12]. At the molecular level, quercetin has been demonstrated to modulate cell signaling pathways and associated molecules, such as p53, phosphatidylinositide-3-kinase (PI3K)/Akt, signal transducer and activator of transcription 3 (STAT3) and epidermal growth factor receptor (EGFR) [8,15]. 

Quercetin could exert antitumor effects by preventing hepatocellular carcinoma LM3 cells from proliferating, migrating and invading, causing cell cycle arrest and apoptosis and stimulating HCC autophagy *via* abrogating the JAK2/STAT3 signaling pathway (by downregulating JAK2 and STAT3) [15]. Quercetin induces mitochondria-mediated apoptosis *via* reducing the Bcl-XL/Bcl-XS ratio, subsequently leading to the activation of caspase-3 and caspase-9 and the translocation of Bax to the mitochondrial membrane in a HepG2 cells [16]. Therefore, quercetin could initiate cell apoptosis and cycle arrest in a dose- or time-dependent manner to suppress the development of most liver cancer cell lines [17]. 

Apoptosis is a programmed process (“a cellular suicide program”) involving the activation of caspases (cysteine proteases) to eliminate superfluous or damaged cells from the organism during development or following cellular stress [18,19]. The transcriptional regulator, p53, acts as a tumor suppressor and can activate and promote apoptosis *via* transcription-dependent and/or -independent mechanisms [20]. During apoptosis and cell growth arrest, p53 integrates stress signals into various antiproliferative responses, blocks cell cycles, promotes apoptosis and maintains the stability of the genome [21]. Disruption of this process facilitates tumor progression and chemoresistance [20]. Quercetin could modulate the apoptotic pathway and promote tumor cell death through stimulating the proapoptotic genes and inhibiting the antiapoptotic genes [18]. Accordingly, p53 likely plays a key role in quercetin’s therapeutic effects against cancers. Additionally, p53 is negatively regulated by the multifunctional transcription factor Yin Yang 1 (YY1), which is expressed in mammalian cells and regulates p53 through direct protein–protein interactions [22]. That is, YY1 overexpression dramatically reduced endogenous p53, whilst loss of YY1 significantly raised p53 level [22,23]. YY1 can regulate the transcription of many genes as a transcriptional activator, repressor or initiator element-binding protein. The biological processes that YY1 is involved in include DNA repair, autophagy, cell differentiation, proliferation and apoptosis. [24]. YY1 plays a crucial role in neurodevelopment, Parkinson’s disease, Alzheimer’s disease, oxidant stress, inflammation, ischemic damage and various cancers (breast cancer, pancreatic cancer, lung adenocarcinoma and colon cancer) [23]. The stability of the p53 protein depends highly on its interactions with YY1. 

In this study, HepG2 cells, human hepatoma cell line, were employed to investigate how quercetin would exert anticancer functions in hepatoma cells and disrupt YY1-p53 interaction. The possible interaction between quercetin and YY1 was further examined by molecular docking, cellular thermal shift assay, UV-Vis absorption spectroscopy, fluorescence spectroscopy, and circular dichroism spectroscopy. This work sheds light on the molecular mechanisms underlying the anticancer function of quercetin.

## 2. Material and Methods

### 2.1. Materials

Quercetin (purity > 98%; CAS: 117-39-5) was purchased from Sigma-Aldrich (St. Louis, MO, USA). Unless otherwise stated, all additional reagents were of analytical quality and bought from Sigma-Aldrich (St. Louis, MO, USA). YY1 protein was obtained via prokaryotic expression and purification according to a published method [25].

### 2.2. Cell Culture

HepG2 cells were obtained from Gaining Biological (Shanghai, China), and were then cultured in fresh minimum essential medium (MEM) (Solarbio, Beijing, China) with 10% fetal bovine serum (FBS) (Solarbio, Beijing, China) and 1% penicillin (100 U/mL)/streptomycin(100 μg/mL) mixture (100 U/mL penicillin; 100 μg/mL streptomycin) (Solarbio, Beijing, China) in a humidified incubator with 5% CO_2_ at 37 °C. The detailed culture steps followed a published procedure [26]. 

### 2.3. Cell Viability Assays

The 3-(4,5-dimethylthiazol-2-yl)-2,5-diphenyltetrazolium bromide (MTT) assay was used to evaluate the cell viability. At a density of 2.0 × 10^4^ cells/mL, HepG2 cells were sown into the 96-well plates at a density of 2.0 × 10^4^ cells/mL and allowed seeding for 24 h. The cells were then exposed to quercetin (final concentration at 5, 10, 20, 30 and 40 μM) for 24, 48 or 72 h, respectively. After the treatments, we rinsed the cells twice using precooled phosphate-buffered saline (PBS, pH = 7.4) and then added 100 μL MTT (Solarbio, Beijing, China) solution (0.5 mg/mL) to each group of cells to incubate for 4 h. After removing the MTT medium, each well was filled with isopycnic DMSO (Solarbio, Beijing, China). The absorbance of each sample was recorded at 570 nm by a multi-detection microplate reader (BioTek Instruments Inc., Winooski, VT, USA). The relative cell viability was calculated based on the proportion of the OD value of a treated group of cells to that of the control group of cells. All the control and treated cells were harvested for further experiments.

### 2.4. Transfection Experiments

The transfection of YY1 overexpression or knockdown was performed using the Lipofectamine^TM^ 2000 Reagent (Invitrogen, New York, NY, USA) according to the manufacturer’s instructions. The plasmids pcDNA3.1-YY1 for overexpression and pLKO.1-puro-shRNA-YY1 for knockdown (both final concentration: 50 nM) were used for each transfection into cells with 2 mL of culture medium in 6-well plates. After transfection for 24 h, the cells were treated with quercetin as described in Section 2.3. Sequences for shRNA were 5′-ACCGGTCATAAAGGCTGCACAAAGATTCAAGAGATCTTTGTGCAGCCTTTATGTTTTTTGAATTC-3′.

### 2.5. Apoptosis Assays

HepG2 cells were seeded and treated as described in Section 2.2 and Section 2.3. Then, they were harvested for an apoptosis assay with the Annexin V-FITC/PI cell apoptosis detection kit (Servicebio, Wuhan, China) according to the manufacturer’s guidelines. Fluorescence shifts were examined by flow cytometry using a BD Accuri C6 Plus Flow Cytometer (BD Biosciences). 

### 2.6. Western Blot Experiments

Cells were lysed using an ice-cold lysis buffer with the protease inhibitor PMSF (Beyotime, Nantong, China), and the concentrations of proteins in the supernatant was determined by the bicinchoninic acid (BCA) method. An amount of 10% (w/t) sodium dodecyl sulphate-polyacrylamide gel electrophoresis (SDS-PAGE) was used to separate protein (30 μg) samples, which were then transferred to polyvinylidene fluoride (PVDF) membranes (Millipore, Bedford, MA, USA). An amount of 5% skim milk in Tris-buffered saline (TBST) (containing 0.1% Tween-20) was used to block the membranes for two hours at room temperature. After three 10 min TBST washes, the resulting membranes were incubated overnight at 4 °C with the following primary antibodies: YY1 (ab109237, 1:5000, Abcam, Waltham, MA, USA), p53 (ab32389, 1:10000, Abcam, Cambridge, MA, USA), HDM2 (ab16895,1:500, Abcam, USA), Bcl-2 (ab196495, 1:500, Abcam, USA), Bax (ab182733, 1:2000, Abcam, Cambridge, MA, USA), and β-actin (ab179467, 1:5000, Abcam, Cambridge, MA, USA). Using a Chemiluminescence Imaging System (Synoptics, Cambridge, UK) with an enhanced chemiluminescence (ECL) Western blotting substrate (Advansta Inc., Menlo Park, CA, USA), the immunoreactive bands were seen and analyzed.

### 2.7. Molecular Docking

YY1 and p53 three-dimensional (3D) structures (Protein Data Bank (PDB) ID: 1UBD and 3Q06, respectively) were obtained from the PDB bank and established with AutoDock Tools. The 3D structure of quercetin was obtained from the PubChem Compound library. The flexible-rigid docking for quercetin and the acceptor protein was performed to examine their possible interaction using the AutoDock 4.0 software. The AutoDock Tools program version1.5.6rcl was used to process the acceptor protein and quercetin to combine non-polar hydrogen atoms, compute Gasteiger partial charges and set rotatable side-chain bonds. The program AutoGrid 4.2 was used to generate the grid box for YY1 docking evaluation (grid spacing, 0.375 Å; size, 72 × 82 × 90 lattice points; grid center, (−2.072, 50.807, 21.43)). The docking calculations were accomplished using Lamarckian Genetic Algorithm with 20 independent runs to obtain the optimal docking conformation (the predominant binding mode). For each protein–ligand pair, the final docking score was taken as the average and the top-scoring docked poses were saved to analyze the binding sites.

The interaction between YY1 and p53 was examined by HDOCK server [27]. The top-scoring docked poses were chosen and further analyzed using PyMol 2.5.0 + openvr software.

### 2.8. Preparation of YY1 Protein and Quercetin Solutions

YY1 protein was dissolved in a phosphate-buffered solution (20 mM K_2_HPO_4_/KH_2_PO_4_, 0.6 M NaCl, pH 7.4) to prepare the YY1 protein stock solution at 10 mg/mL. The PBS was used to dilute the quercetin that had been dissolved in DMSO to different concentrations. 

### 2.9. The Cellular Thermal Shift Assay (CETSA)

In 6-well plates, the cells were sown at a density of 2 × 10^4^ cells/mL and digested with trypsin, and subsequently washed twice by PBS. The digested cells were washed twice with the phosphate-buffered solution, then mixed with the phosphate-buffered solution containing phosphatase inhibitor and protease inhibitor. Such a cell suspension was subjected to a freeze–thaw cycle three times (i.e., treatment with liquid nitrogen then a water bath at 37 °C), to lyse cells. The resulting sample was centrifuged (20,000× *g*, 4 °C 20 min). The obtained supernatant was divided into portions, which were mixed, respectively, with equal volumes of DMSO and quercetin (30 μM). These mixtures were left to stand at room temperature for 30 min, and each of these mixtures was divided into portions for different thermal treatments (from 40 °C to 76 °C for 3 min) using a thermal cycle meter. The YY1 protein contents in these heated samples were subjected to Western blot analysis. YY1 in DMSO was used as an internal control.

### 2.10. UV-Vis Absorption Spectroscopy for Examining YY1-Quercetin Mixtures

The UV–Vis absorption spectra were measured by a multi-function microplate reader (SpectraMax^®^ M5, Shanghai, China). Mixing the YY1 protein solution and one of the quercetin solutions with different concentrations to obtain YY1-quercetin mixtures, the final constant YY1 concentration was 0.1 mg/mL and final quercetin concentrations were 0, 2, 4, 6, 8 and 10 µg/mL, respectively. The UV–Vis absorption spectra of the resulting YY1-quercetin mixtures at 25 °C were acquired at 250 and 330 nm using a multi-function microplate reader (SpectraMax^®^ M5, Shanghai, China). The spectra of the phosphate-buffered solution were recorded between 250 and 330 nm at 25 °C. Additionally, the spectra of PBS without and with quercetin were used as the controls for subtraction.

### 2.11. YY1-Quercetin Interaction Revealed by Fluorescence Spectroscopy

The fluorescence spectra were recorded using a spectrofluorometer (Horiba, Kyoto, Japan, USA) and 10 mm fluorescence quartz cuvettes. The YY1 protein solution, quercetin solution, and YY1-quercetin mixtures were the same as those prepared in Section 2.10. The emission spectra were captured at 298 K between 300 and 460 nm with the excitation wavelength set at 280 nm. Excitation and emission slit widths were both set at 10 nm. The PBS was used as a blank (background signal) to correct the fluorescence intensity curves. The spectra of quercetin alone at different concentrations were used as the controls for subtraction from the raw spectra of the YY1-quercetin test samples with the corresponding quercetin concentrations.

The fluorescence quenching induced by quercetin was further examined using the Stern–Volmer Equation [28]

F_0_/F = 1 + K_sv_[Q] = 1 + K_q_τ_0_[Q]

where F_0_ and F are the protein fluorescence intensities at the emission maximum without and with quercetin; K_sv_ is the Stern–Volmer quenching constant; [Q] is the concentration of quencher; K_q_ is the biomolecular quenching rate constant; τ_0_ is the average lifetime of the biomolecule without quencher (τ_0_ = 10^−8^ s).

The double log Stern–Volmer equation log[(F_0_ − F)/F] = logK_a_ + nlog[Q] allows the determination of the binding constant (K_a_) and number of binding sites (n).

### 2.12. YY1-Quercetin Interaction Revealed by Circular Dichroism (CD)

CD spectra of YY1 protein solutions with or without quercetin (final YY1 concentration, 0.02 mg/mL; final quercetin concentration, 0.5 μg/mL) were obtained using a Chriascan CD spectropolarimeter (Applied Photophysics Inc., Leatherhead, Surrey, UK). The CD spectra were acquired from 200 to 260 nm at 25 °C and the scan speed was set at 100 nm/min. The final spectrum of an analyzed sample was the average of the spectra resulting from three consecutive measurements. Background spectra of quercetin alone at various concentrations were subtracted from the raw spectra of the YY1-quercetin test samples with the corresponding quercetin concentrations. The Chriascan spectrometer’s protein secondary structure calculation software (CDNN program) was used to calculate the protein secondary structure.

### 2.13. Statistical Analysis

Each experiment was performed in triplicate, with the data being presented as “mean ± standard deviation (SD)”. The Statistical Package for the Social Science (SPSS) statistical software version 18.0 (SPSS, Chicago, IL, USA) was used to conduct the statistical analyses. The statistical significance was determined by one-way analysis of variance (ANOVA) followed by Duncan’s multiple range test. If *P* was less than 0.05, differences were considered significant.

## 3. Results

### 3.1. Quercetin Inhibited Cell Viability in HepG2 Cells

**Figure 1:**
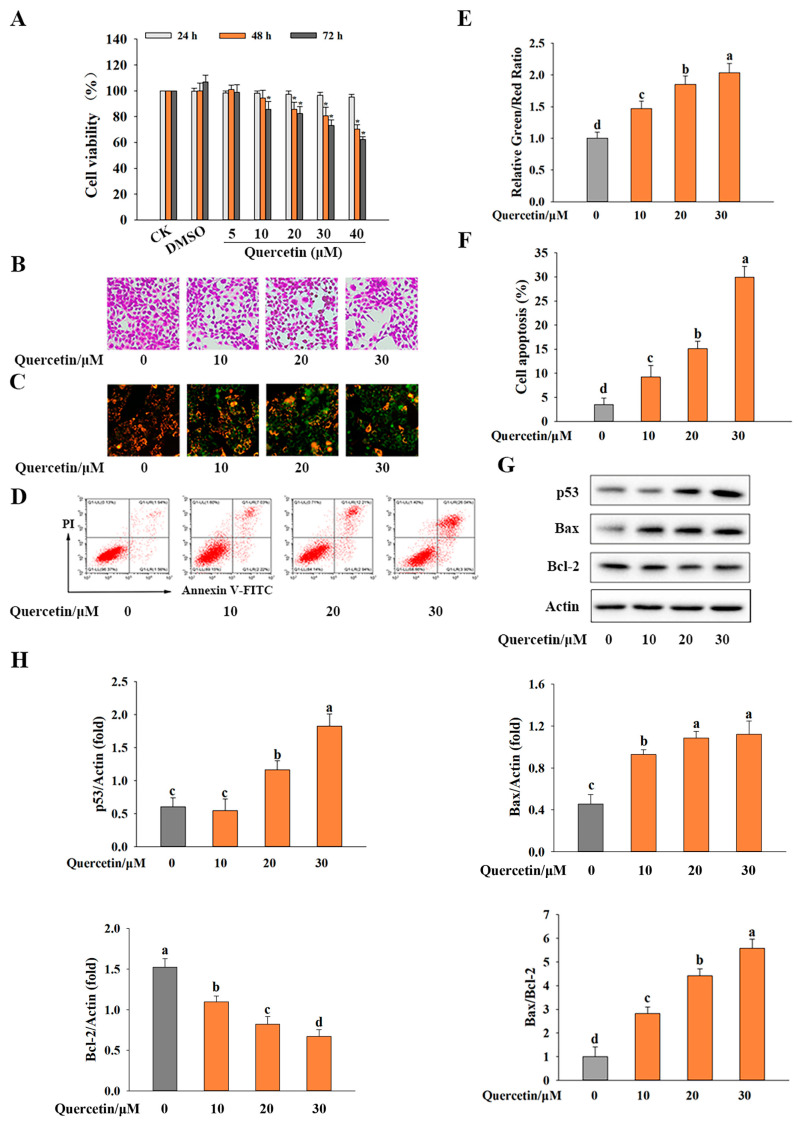
Quercetin reduced the cell viability in HepG2 cells. (**A**) The cell viability after quercetin (5, 10, 20, 30 or 40 μM) treatment for 24, 48 or 72 h in HepG2 cells, with DMSO used as a solvent control; (**B**) the crystal violet stained cells before and after quercetin (10, 20 or 30 μM) treatment for 72 h; (**C**) the mitochondrial membrane potential for the cells stained with 5,5,6,6′-tetrachloro-1,1′,3,3′ tetraethylbenzimi-dazoylcarbocyanine iodide (JC-1); (**D**) apoptosis level revealed by flow cytometry; (**E**) relative mitochondrial membrane potential (green/red fluorescence intensity ratio); (**F**) apoptosis percentage of HepG2 cells; (**G**) protein expressions of p53, Bax and Bcl-2; (**H**) relative protein expression of p53, Bax, Bcl-2 and Bax/Bcl-2 ratio. β-Actin was used as a loading control. Data are presented as “mean ± standard deviation” (*n* = 3). Data were statistically analyzed by one-way ANOVA: (**A**) * (*p* < 0.05) versus the control group; (**E**–**H**) distinct letters (a–d) show significant (*p* < 0.05) difference between different groups.

As shown in Figure 1A, different concentrations of quercetin (0, 5, 10, 20, 30, 40 μM) were applied to HepG2 cells for 24, 48 and 72 h, respectively, and DMSO (0.1%) was used as a solvent control. All the DMSO groups showed no cytotoxic effect on HepG2 cells. Quercetin reduced the cell viability of HepG2 in a dose- and time-dependent manner. However, the cell viability of the cells treated with quercetin at 5–20 μM for 24 h was essentially identical to that for the control (CK), indicating that these quercetin treatments exhibited no cytotoxicity to HepG2 cells. This finding was consistent with other experimental published results [29]. The treatments with quercetin (10, 20 or 30 μM) for 72 h significantly reduced the cell viability (i.e., decreased by 85.68%, 82.45% and 73.16%, respectively), and thus these treatments were selected for subsequent experiments. 

The differences in the number of crystal violet-stained cells (Figure 1B) showed that the treatment with quercetin (10, 20 or 30 μM) for 72 h reduced the viability of HepG2 cells, especially the treatment with 30 μM. These treatments also significantly decreased the mitochondrial membrane potential (Figure 1C,E). This result suggests that quercetin at 10, 20 or 30 μM could induce apoptosis of HepG2 cells, as reduction of the mitochondrial membrane potential is a distinctive change during cell apoptosis [30,31]. The flow cytometry experiments showed that quercetin induced apoptosis in a dose-dependent manner (Figure 1D), with the apoptosis in HepG2 cells being 9.25%, 15.15% and 29.9%, respectively, for treatment with quercetin at 10, 20 or 30 μM (Figure 1F). This result supported the study’s findings on cell viability, suggesting that quercetin could induce apoptosis and suppress cell proliferation in HepG2 cells. 

### 3.2. Quercetin Induced Apoptosis by Regulating p53-Mediated Signaling Pathway in HepG2 Cells

One of the most important functions of p53 is the induction of apoptosis. As shown in Figure 1G,H, the pre-treatment with quercetin at 30 μM substantially increased the protein expressions of p53 (by 3.0 fold) and Bax (by 1.4 fold) and decreased Bcl-2 protein expression (by 0.4-fold) (*p* < 0.05). Moreover, quercetin greatly increased the Bax/Bcl-2 ratio. These results indicated that quercetin exerted an anticancer effect in HepG2 cells through upregulating the p53-related apoptotic pathway. 

### 3.3. YY1 and Its Role in Quercetin-Induced Apoptosis of HepG2 Cells

**Figure 2:**
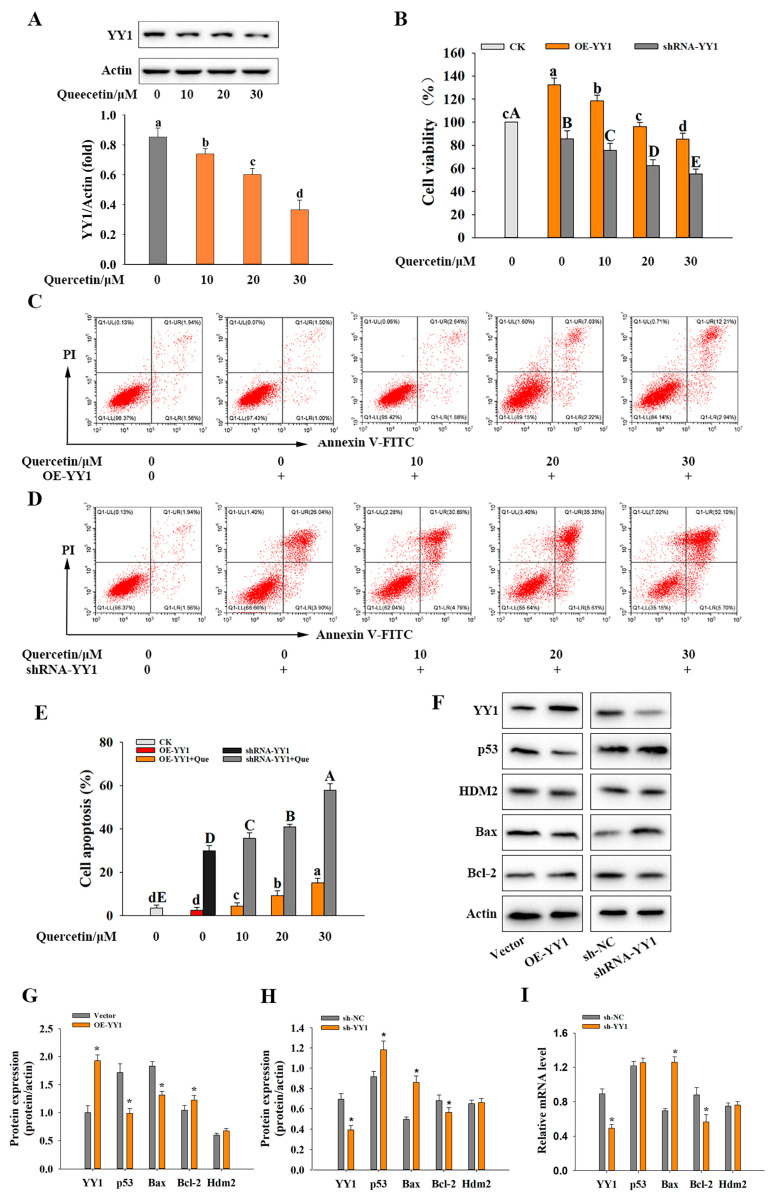
YY1 expression and its role in quercetin-induced apoptosis of HepG2 cells: (**A**) effect of quercetin on YY1 protein expression in HepG2 cells; (**B**) the cell viability after transfection of OE-YY1 or shYY1. The pcDNA3.1-YY1 (for overexpression YY1; OE-YY1) or shRNA-YY1 (shYY1; for knockdown YY1) was transfected for 24 h; (**C**,**D**) the apoptosis of the HepG2 cells with OE-YY1 (**C**) or shYY1 (**D**) examined by flow cytometry; (**E**) the apoptosis rates for the HepG2 cells subjected to different treatments; (**F**) the protein expressions of YY1, p53, HDM2, Bax and Bcl-2 in the HepG2 cells with OE-YY1 or shYY1; (**G**,**H**) relative protein expressions of YY1, p53, HDM2, Bax and Bcl-2 in the HepG2 cells with OE-YY1 (**G**) or shYY1 (**H**); (**I**) relative mRNA expressions of YY1, p53, HDM2, Bax and Bcl-2 in the HepG2 cells with shYY1. Actin was used as a loading control. Data are presented as “mean ± standard deviation” (*n* = 3). Data were statistically analyzed by one-way ANOVA: distinct letters (a–d) and (**A**–**E**) indicate significant (*p* < 0.05) difference between the different groups; (**G**–**I**) * (*p* < 0.05) versus the control group.

Since YY1 can negatively regulate p53 via direct physical protein–protein interactions [22], and herein quercetin’s anticancer action was found through upregulating the p53-related apoptotic pathway (Section 3.2), the function of YY1 in the quercetin-induced apoptosis of HepG2 cells was also examined in this study. Quercetin significantly decreased YY1 protein expression (by 0.4-fold, approximately, at a quercetin concentration of 30 μM) in HepG2 cells compared to the control. To compare the biological functions of YY1 in HepG2 cells without or with quercetin, YY1 was overexpressed and knocked down, respectively, in two separate experiments. As shown in Figure 2B–E, the HepG2 cell viability was significantly increased, and the apoptosis rate was decreased after YY1 overexpression when compared with the control. The use of quercetin counteracted the YY1 overexpression-induced decrease in apoptosis rate, and instead raised (in a dose-dependent manner) the apoptosis rate to the level that was considerably greater than the control (Figure 2E). In another set of experiments, the knockdown of YY1 reduced the cell proliferation of HepG2 cells, with the corresponding apoptosis rate increasing by 26.44% in contrast with the control group (Figure 2E). The presence of quercetin promoted the HepG2 cells’ apoptosis and further enhanced YY1 knockdown-initiated increase in apoptosis, leading to an apoptosis rate of 57.8% at a quercetin concentration of 30 µM (Figure 2E). These results indicated that YY1 could participate in quercetin-induced apoptosis of HepG2 cells, and both the expression level of YY1 and quercetin concentration were crucial for the apoptosis of HepG2 cells.

In this work, YY1 overexpression resulted in a substantial decrease in p53 protein expression, and significant changes in p53 downstream apoptosis-related proteins, including the downregulation of Bax (a proapoptotic protein) and upregulation of Bcl-2 (an antiapoptotic protein) (Figure 2F). Conversely, the knockdown of YY1 significantly increased p53 expression, upregulated Bax expression and downregulated Bcl-2 expression, which explained the promotion of apoptosis caused by the knockdown of YY1 in HepG2 cells. However, the protein expression of HDM2 (the E3 ligase human double minute 2, an important negative regular of p53) did not alter appreciably when YY1 was overexpressed or knocked down (Figure 2F,I). Furthermore, the knockdown of YY1 did not significantly change the p53 mRNA level but upregulated Bax mRNA expression (Figure 2). This suggested that YY1 may regulate p53 expression by protein–protein interactions rather than at the transcription level.

### 3.4. The Interaction between Quercetin and YY1

#### 3.4.1. Molecular Docking Showed the Interactions between Quercetin and YY1

**Figure 3:**
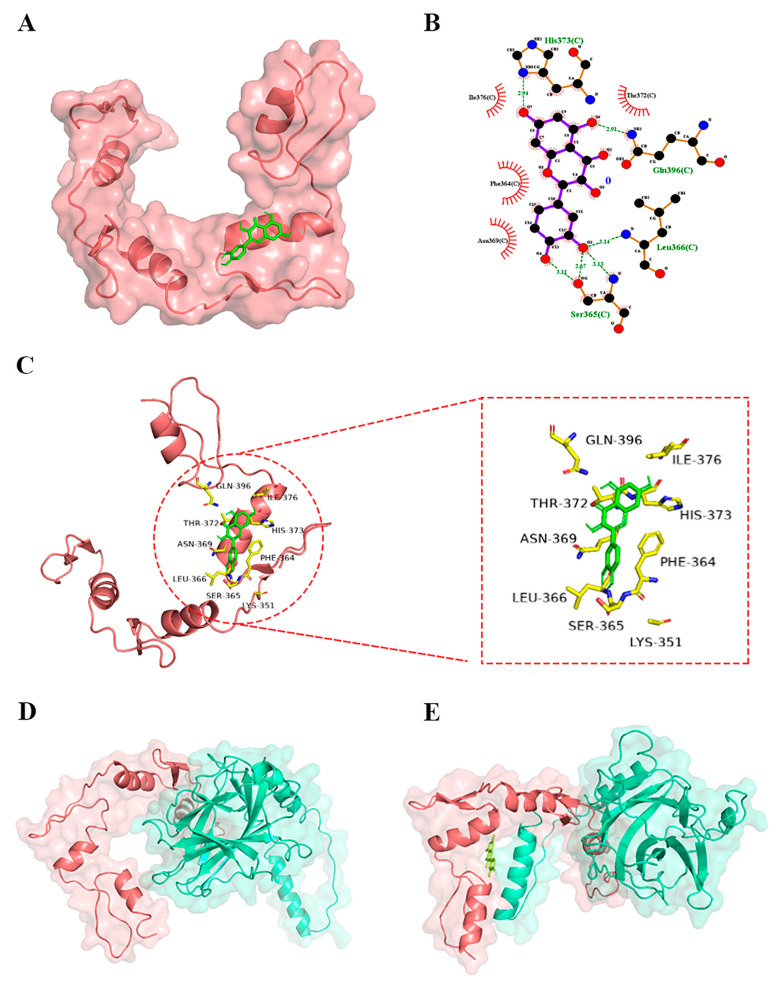
The surface (**A**), 2D (**B**) and 3D (**C**) docking poses of YY1 with quercetin. (**D**,**E**) The docking pose of YY1 with p53 without (**D**) or with (**E**) quercetin. The red protein model is YY1, the cyan protein model is p53, and the green stick is quercetin.

The 3D, 2D and surface docking poses of quercetin with YY1 protein (Figure 3) showed that quercetin interacted with the active sites of YY1 through hydrogen bonding and hydrophobic interactions, leading to a docking score (binding energy) of −6.73 kcal/mol. The comparison of this score with the binding energy between quercetin and p53 (−4.96 kcal/mol) revealed that quercetin had a higher binding affinity for YY1 than for p53. 

As shown in Figure 3, quercetin, as hydrogen donors or hydrogen acceptors, formed hydrogen bonds via its hydroxyl groups and oxygen atoms with some amino acids of YY1 (i.e., Ser365, Leu366, Gln396 and His373). In the meantime, quercetin interacted with these amino acid residues of YY1 protein, Thr372, Ile 376, Phe364 and Asn369, via hydrophobic interactions. Previous research reported that YY1 interacted with p53 via direct protein–protein interactions in the amino acid 142–224 and 331–414 regions [22]. Therefore, the binding sites for quercetin on YY1 were mainly located in the region of YY1 that interacted with p53, suggesting that quercetin probably directly bound to YY1, thereby disrupting the YY1-p53 interaction and then activating p53 and downstream apoptosis-related proteins. 

The molecular docking between YY1 and p53 by HDOCK revealed that their docking energy was −264.68 kcal/mol. When quercetin bound to YY1, the docking energy between YY1 and p53 became −224.28 kcal/mol, suggesting that the binding of quercetin with YY1 might suppress the YY1-p53 interaction. Additionally, the binding of quercetin to YY1 affected the binding pose and sites of p53 to YY1 (Figure 3D,E). Therefore, the binding of quercetin with YY1 might inhibit the YY1-p53 interaction to activate the p53-related apoptosis pathway.

In order to further examine the exact interaction between quercetin and YY1, the residues Ser365, Leu366, Gln396 and His373 of native YY1 were individually mutated into alanine (the resulting YY1 was named mutant-YY1). After mutation, the docking energy of quercetin was reduced from −6.73 kcal/mol to −4.32 kcal/mol. Taken together, quercetin might interact with YY1 at the same binding sites for p53, thereby increasing the level of available p53. However, the interaction between quercetin and YY1 needs to be examined further.

#### 3.4.2. The Direct Binding of Quercetin to YY1 Revealed by CETSA 

**Figure 4:**
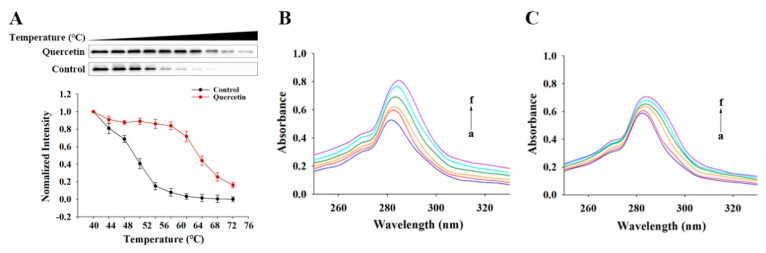
(**A**) Quercetin increased the thermal stability of YY1 in cell lysates by the temperature-dependent cellular thermal shift assay. YY1 in DMSO as the control, and YY1 in the presence of quercetin in DMSO is termed “Quercetin” herein; (**B**,**C**) UV–Vis absorption spectra of YY1 (**B**) and mutant-YY1 (**C**) with and without quercetin. The concentration of YY1 or mutant-YY1 protein was 0.2 mg/mL. The test samples with quercetin at concentrations 0, 2, 4, 6, 8 and 10 μg/mL correspond to lowercase letters from a to f.

The quercetin-induced thermal stabilization of target protein YY1 was examined by CETSA to study the interaction between quercetin and YY1 in a cellular context. According to Figure 4A, the protein stability of YY1 in the presence of DMSO decreased greatly with an increase in temperature over 40–76 °C (especially 40–60 °C). The presence of quercetin in DMSO counteracted the thermally induced damage to YY1 and increased the thermal stability of YY1 over 56–76 °C (especially 56–64 °C). Therefore, there was a direct interaction between quercetin and YY1, by which the protein stability of YY1 was greatly improved.

#### 3.4.3. UV–Vis Absorption Spectra of Quercetin–YY1 Systems

Protein structural changes are commonly investigate using UV-Vis absorption spectroscopy after they form complexes with small molecules, based on the absorption near 280 nm derived from the protein’s aromatic amino acid residues (Tyr, Trp and Phe) [32]. Compared with the YY1 system alone, the intensities of the absorption around 282–286 nm for the YY1-quercetin systems increased significantly (maximum absorbance increased by 57.06%), and their maximum absorption peaks exhibited significant red shifts (Figure 4B). The obvious red shift of maximum peak position was also a result of the quercetin addition (from 285 to 290 nm). Figure 4C showed the absorption spectra of mutant YY1 with/without quercetin. Compared with the mutated YY1 alone, the presence of quercetin along with mutated YY1 also led to concentration-dependent absorbance increases and peak red shifts. However, a small absorbance increment (e.g., maximum absorbance increased by 26.12%) was found for the mutated YY1-quercetin than for the YY1-quercetin. A smaller maximum absorption was also found for the mutated YY1 alone than for the native YY1 alone. Such differences between the native YY1 systems and the mutated YY1 systems indicated that the mutation caused changes in YY1 protein structure. It is worth noting that quercetin also has strong UV–Vis absorption around 280 nm. Therefore, the current UV–Vis absorption method could not distinguish whether the absorbance increase detected in the YY1-quercetin systems resulted from the changes in the YY1 protein or was induced by quercetin itself.

#### 3.4.4. Fluorescence Spectroscopy of YY1 Induced by Quercetin

**Figure 5:**
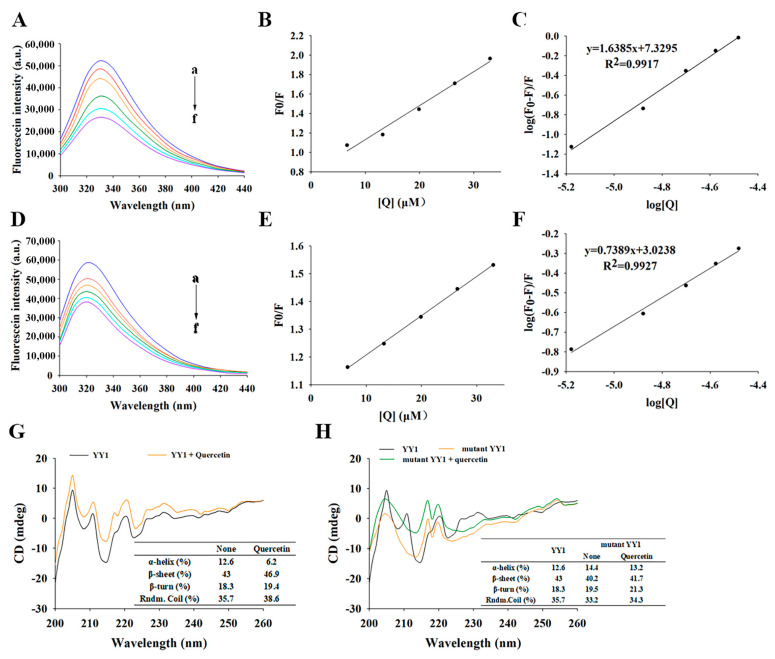
Fluorescence emission spectra (λex = 280 nm; 298 K) of native YY1 (**A**) and mutant YY1 (**D**) without or with quercetin. The concentration of YY1 or mutant YY1 was 0.2 mg/mL, and the quercetin concentration was 0, 2, 4, 6, 8 or 10 µg/mL (corresponding to a to f); Stern–Volmer plots (**B**,**E**) and derived logarithmic plots (**C**,**F**) corresponding to the quenching of native YY1 (**B**,**C**) or mutant YY1 (**E**,**F**) fluorescence by quercetin at 298 K; (**G**,**H**) circular dichroism spectra of native YY1 (**G**; 0.02 mg/mL) and mutated YY1 (**H**; 0.02 mg/mL) without or with quercetin (0.5 μg/mL). Rndm.Coil represents random coils.

Fluorescence spectroscopy is regularly used to study the interaction of small molecules with proteins, based on the changes in the fluorescence of aromatic chromophores in proteins, e.g., tryptophan (Trp) and tyrosine (Tyr), which can produce endogenous fluorescence at an excitation wavelength of 280 nm [33]. The fluorescence emission spectra of YY1 without or with quercetin are shown in Figure 5A. When the excitation wavelength is 280 nm, YY1 showed its characteristic emission peak at 330 nm. The quenching of YY1 fluorescence increased with an elevated quercetin concentration, with the decrease in fluorescence intensity induced by quercetin up to 49.10%. 

The quenching constant K_q_ for YY1 with quercetin was calculated as 2.92 × 10^12^ M^−1^ S^−1^, based on the linear plots of F_0_/F versus [Q] (Figure 5B), which was about 100 times the highest scatter collision quenching constant of dynamic quenching (2.0 × 10^10^ M^−1^ S^−1^) [34,35]. Therefore, static quenching rather than dynamic collision was responsible for quenching the YY1 fluorescence in response to quercetin [36]. The double log plots (Figure 5C) revealed that the number of binding sites n for YY1-quercetin was 1.63 and the binding constant K_a_ was 2.14 × 10^7^ M^−1^. These results suggested that quercetin had a rather high affinity for YY1 [9].

Interestingly, if the mutant YY1 was used in the place of the native YY1, the fluorescence intensity decreased by 34.71% and a slight blue shift was observed from 322 nm to 318 nm (Figure 5D). These results indicate that the presence of quercetin could still cause significant quenching of mutant YY1 fluorescence, which probably resulted from quercetin-induced changes in the microenvironment of the mutant YY1, e.g., altered the conformation of mutant YY1, increased the exposure of some buried Trp or Tyr residues while making their surrounding microenvironments more polar, and facilitated charge redistribution during excitation. These microenvironmental changes induced by the presence of quercetin also took place in the case of native YY1. When quercetin interacted with mutant YY1, the K_q_ was 1.34 × 10^12^ M^−1^ S^−1^ and the K_a_ decrease was 1.06 × 10^3^ M^−1^ (Figure 5E,F), both of which were less than half of those of native YY1. Taken together, the mutation of YY1 in this study led to a considerable impairment of the binding affinity between YY1 and quercetin, and the YY1-quercetin interactions mainly included the direct binding of quercetin to YY1 and some indirect interactions between YY1 and quercetin.

#### 3.4.5. Quercetin-Induced Changes in the Conformation of YY1 Protein Revealed by Circular Dichroism (CD) Spectroscopy

In this study, the secondary structure changes of native YY1 were examined by CD in the far UV region (Figure 5G). The interaction of YY1 with quercetin induced the decrease in *α*-helix content from 12.6% to 6.2%, and the increases in the *β*-sheet content from 43% to 46.9%, *β*-turn content from 18.4% to 19.4% and random coils content from 35.7% to 38.6%. The result indicated that the interaction between YY1 and quercetin caused partial stretching of YY1′s peptide chains and made the YY1-quercetin complex adopt a more extended and looser conformation. The CD spectrum of mutated YY1 (Figure 5H) differed significantly from that of the native YY1 (Figure 5G), indicating that the mutation of YY1′s binding sites for quercetin led to significant changes in YY1′s secondary structures. When quercetin interacted with the mutant YY1 protein, mutant YY1′s *α*-helix content decreased from 14.4% to 13.2%, *β*-sheet content increased from 40.2% to 41.7%, *β*-turn content decreased from 19.5% to 21.3%, and random coils content increased from 33.2% to 34.3%. Compared to the interaction between the native YY1 and quercetin, the interaction between the mutant YY1 and quercetin caused slightly less unfolding and loosening effects on the protein, though still induced significant changes in the protein’s secondary structures. This result was consistent with the finding obtained by fluorescence spectroscopy, that besides the direct binding between YY1 and quercetin, some non-binding interactions also occurred between quercetin and the native/mutant YY1 (involving other amino acid residues rather than the identified binding sites) (Section 3.4.4). The results obtained herein confirmed that quercetin might directly bind to the YY1 protein at His373, Glin396, Leu366 and Ser365, and their interactions might alter dynamically with the changes in YY1′s conformation.

## 4. Discussion

As a flavonoid with the anticancer effect, quercetin has been reported to trigger apoptosis and suppress proliferation and migration in multiple human cancer cell lines, including HCC cells [11,12,15]. In this study, quercetin (at 10, 20 or 30 μM) promoted cell apoptosis and decreased the cell survival of HepG2 in a dose- and/or time-dependent way. Lambert and Yang believed that such high quercetin concentrations cannot be obtained in vivo from many target tissues [37]. 

In this study, although it seems that the higher quercetin concentration in vitro experiments results in some limitations, there has been increasing effort to improve the bioavailability of quercetin through increasing its aqueous solubility, improving its stability along the gastrointestinal tract, slowing down its metabolism and prolonging its biological half-life in recent years [38]. It has been developed that a wide range of delivery systems help quercetin to overcome its limited bioavailability, including phytosomes, micelles, polymeric nanoparticles, multifunction micellar nanoparticles, emulsions, liposomes, and other nanostructured lipid carriers [39]. Compared to the free quercetin (25 mg/kg bodyweight in rats), the half-life of quercetin in rats administered with liposomal quercetin was six times greater, and the liposomal quercetin could accumulate well in the liver, brain and heart with the contents of 1.0, 2.7, 0.5 μg/g tissue, respectively, 24 h after administration [40]. Oligochitosan-de-esterified yuzu peel pectin-based hydrogel beads were found capable of entrapping quercetin efficiently to improve its stability in simulated gastric and intestinal fluids [41]. Xiong et al. constructed a novel hyaluronic-acid-based quercetin nanoformulation to allow high drug loading for targeted tumor therapy [42]. The phenylboronic-acid-conjugated zinc oxide nanoparticles loaded with quercetin facilitated the delivery of quercetin targeting sialic acid over-expressed cancer cells to induce cell apoptosis in human breast cancer cells [43]. Therefore, it is possible to enhance the effective transport and delivery of quercetin to the target tissue (including the tumor tissue) thereby increasing the local concentration of quercetin.

According to this study, quercetin exerted an anticancer effect in HepG2 cells *via* activating the p53-related apoptotic pathway, greatly increasing both p53 protein expression and the Bax/Bcl-2 ratio. p53 has been described as a sensitizer and an activator of apoptosis, as it can transcriptionally activate the expressions of Bax and Bcl-2 [21]. The p53 as a guide to apoptosis can activate apoptosis *via* transcription-independent mechanisms and promote apoptosis and transcription-dependent processes. Disruption of p53-induced apoptosis accelerates tumor progression and chemotherapy resistance [44]. This result supported the notion that quercetin can trigger apoptosis in a variety of cancer cells through modulating the expressions of apoptotic markers [45].

YY1 is a versatile transcription factor determining gene expression [46,47] as well as a negative regulator of p53 to modulate tumorigenesis [22,48]. We discovered that YY1 was crucial in quercetin-induced apoptosis in HepG2 cells because YY1 overexpression increased cell survival and decreased the apoptosis rate, but YY1 knockdown had the opposite effect. The treatment of quercetin promoted the apoptosis of HepG2 cells by inhibiting YY1 to further enhance YY1-knockdown-initiated increase in apoptosis. 

As a negative regulator of p53, YY1 overexpression decreased p53 expression, downregulating downstream Bax and upregulating Bcl-2 expression, while the knockdown of YY1 promoted p53 expression and changed downstream apoptosis-related protein levels. However, regardless of whether YY1 was knocked-down or overexpressed, the protein expression of HDM2 remained essentially unchanged. While HDM2 has been recognized as a p53-specific E3 ubiquitin ligase and the HDM2-p53 loop is believed to be important for modulating p53 expression and activity [49,50], a recent study reported that HDM2 expression on the membrane of cancer cells was p53-independent but not on the membrane of normal cells [51]. Therefore, neither YY1 overexpression nor knockdown altered the protein expression of HDM2 in the HepG2 cells in this work. Interestingly, YY1 knockdown did not greatly affect the mRNA level of p53 but upregulated mRNA expression of Bax. It was possible that YY1 regulated the p53 expression through the YY1-p53 direct physical interactions rather than transcription regulation in HepG2 cells [52]. In melanoma cell lines, YY1 knockdown was previously found to increase the apoptosis rate, reduce the invasion, migration and proliferation of melanoma cell lines, and activated p53 signaling pathway though disrupting the HDM2-p53 interaction then stabilizing and activating p53 [52]. The findings of this study were in line with those of other studies: quercetin induced apoptosis of HepG2 cells and inhibited proliferation probably through interacting with YY1 to disrupt YY1-p53 interaction.

By using molecular docking, the interaction between quercetin and YY1 was examined. Quercetin bound to YY1 with a higher binding affinity through hydrogen bonding and hydrophobic interactions at Ser365, Leu366, Gln396, His373, Thr372, Ile 376, Phe364 and Asn369. The molecular docking between YY1 and p53 in the presence or absence of quercetin confirmed that quercetin could interact with YY1 to disrupt YY1-p53 interaction, inhibiting the proliferation and inducing apoptosis in HepG2 cells. 

The further direct binding of quercetin to the YY1 protein was confirmed by the cellular thermal shift assay, UV-Vis absorption spectroscopy, fluorescence spectroscopy and CD spectroscopy in addition to molecular docking. The native YY1 protein and mutant YY1 protein (the binding sites of quercetin were mutated) were used to help elucidate the binding sites of quercetin to YY1. These results demonstrated that quercetin directly bound to YY1 protein at His373, Glin396, Leu366 and Ser365 and resulted in the significant changes in YY1′s secondary structures. Besides the direct binding between quercetin and YY1, other types of interactions also took place via other amino acid residues (rather than those identified binding sites for quercetin). These other types of YY1-quercetin interactions would alter dynamically with YY1′s conformational changes. Previous research revealed that quercetin could bind to tyrosinase to inhibit tyrosinase’s activity to suppress enzymatic browning in food [53]. The phenolic compounds in berry and citrus can be interacted directly with the dimerization domains of hepatocyte nuclear factor-1*α* (HNF-1*α*, a TF supporting the transcription of proteins linked with type II diabetes) [54]. Additionally, anthocyanins and flavones molecules with sufficient oxygen or glycosides can bind to key amino acids (Ser363, Ser508, Ser555, and Ser602) on Kelch-like ECH-associated protein 1 (Keap1), which are usually bound to Nrf2, to disrupt the protein–protein interaction between Keap1and Nrf2 [55]. Therefore, it is not surprising that quercetin bound directly to YY1 at the binding sites for p53, such as Ser365, Leu366, Gln396, and His373, disrupting the YY1-p53 interaction to activate p53 and downstream apoptosis-related proteins. 

Furthermore, oxidative stress is a major factor in the occurrence and development of cancer. Quercetin, as a natural antioxidant widely existing in fruits and vegetables, can resist oxidative stress damage by scavenging active oxygen free radicals, enhancing antioxidant enzyme activity, improving mitochondrial function and inhibiting cell apoptosis. Therefore, quercetin plays an antioxidant role by regulating several signaling pathways, including Nrf2, MAPK, p53 and NF-κB [56,57,58]. In this study, quercetin promoted cancer cells apoptosis and suppressed proliferation in HepG2 cells through directly interacting with YY1, upregulating p53 and apoptosis-related proteins. The high expression of YY1 is in favor of antioxidant effects in normal nerve cells, despite the fact that it is strongly expressed in a variety of cancers and necessary for tumor formation. In response to oxidative stress, YY1 could enhance Nrf2 transcriptional activity in mouse neurons to improve antioxidative response [59]. It is possible that the regulation of quercetin to YY1 could enhance the synergistic interaction between Nrf2 and YY1, resulting in the improvement of the antioxidant effect. 

Therefore, this work demonstrates the potential of YY1 as a therapeutic target of flavonoids and provides mechanistic insights into quercetin’s anticancer action (i.e., quercetin induced YY1/p53-mediated apoptosis through interacting directly with YY1 and modifying YY1 protein conformation to disrupt YY1 protein–p53 protein interactions).

## 5. Conclusions

Quercetin induced apoptosis and inhibited cell proliferation of human hepatoma cells (HepG2) *via* downregulating the transcription factor YY1, upregulating the tumor suppressor p53 and the downstream Bax, and downregulating Bcl-2. The anticancer actions of quercetin in HepG2 cells may be accomplished through quercetin’s direct binding of the binding sites on the transcription factor YY1 (located within the binding region for p53), along with quercetin-induced alterations in YY1′s conformation. All changes in YY1 disrupted the YY1-p53 interaction, causing HDM2-mediated p53 polyubiquitination and p53 activation, eventually triggering and promoting the apoptotic signaling pathway. Based on the similarity of the structure, rutin, isoquercetin and other flavonoids as well as quercetin derivative/metabolites may also directly interact with YY1 to disrupt the YY1 protein--p53 protein interaction, thereby performing cell apoptosis and a subsequent anticancer effect. Further research is needed to investigate the interaction of flavonoids with the YY1 protein as well as their binding sites. The structure–activity relationship between flavonoid structure difference and anticancer effect is also worth further study.

## Data Availability

The other data that support the findings of this study are available in the Supplementary Material of this article.

## Supplementary Materials

The following supporting information can be downloaded at: https://www.mdpi.com/article/10.3390/metabo13020229/s1. Figure S1. shows the whole blot after cutting membrane at molecular weight 65 kDa, 35 kDa and 15 kDa for p53(53kDa), YY1(45kDa), Actin(42kDa), Bax(21kDa)and Bcl-2(26 kDa). These reprent western blot analysis shown in Fig.land Fig.2A. Since we only added one molecular marker each time in western blot and cut the membrane to many different target bands, we failed to show the complete mecular marker in each band. Please consider our situation. Figure S2. shows the whole blot after cutting membrane atmolecular weight 100 kDa, 75 kDa, 65 kDa, 35 kDa and 15 kDa for HDM2(90 kDa),p53(53kDa)YY(45kDa)Actin(42kDa)Bax(21kDa)and Bc1-2 (26 kDa). These reprent western blot analysis shown in Fig.2F Since weonlyadded one molecular marker each time in westen blot and cut the membrane to many different target bands,we failed to show the complete mecular marker in each band. Please consider our situation.
