# Supplementary material for: Quercetin Induces Apoptosis in HepG2 Cells via Directly Interacting with YY1 to Disrupt YY1-p53 Interaction
Source: Metabolites. 2023 Feb 3;13(2):229. doi: 10.3390/metabo13020229 (PMC9968089; doi:10.3390/metabo13020229)
Supplement: Supplementary file 1 [file metabolites-13-00229-s001.zip › metabolites-2160472-supplementary.pdf]

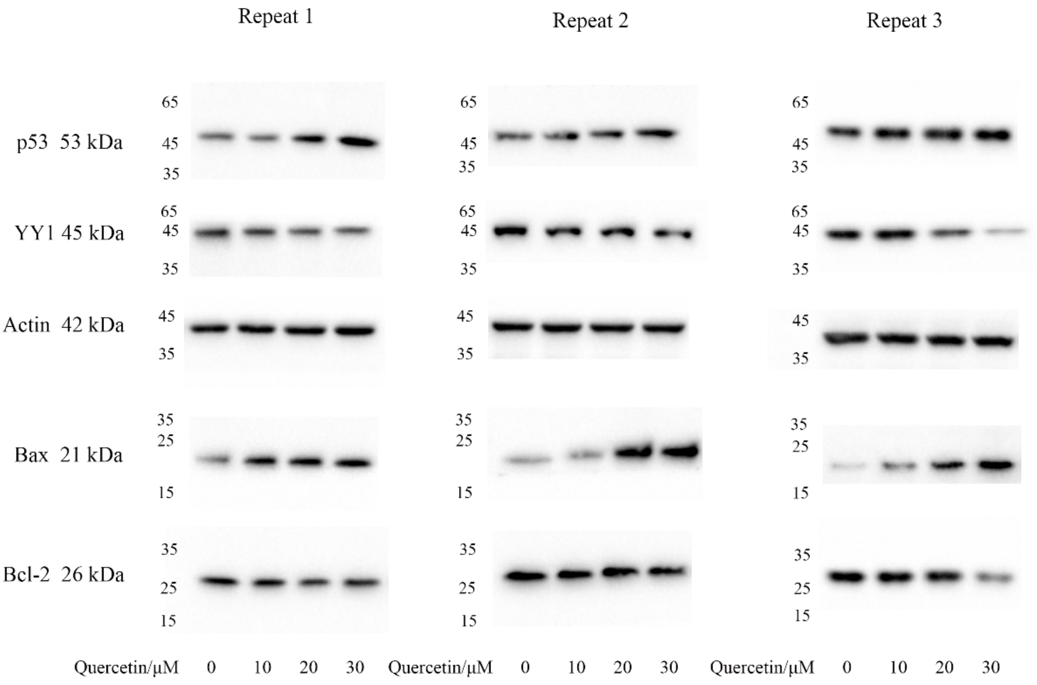

Figure S1 shows the whole blot after cutting membrane at molecular weight 65 kDa, 35 kDa and 15 kDa for p53 (53 kDa), YY1 (45 kDa), Actin (42 kDa), Bax (21 kDa) and Bcl-2 (26 kDa). These reprent western blot analysis shown in Fig.1and Fig. 2A.

Since we only added one molecular marker each time in western blot and cut the membrane to many different target bands, we failed to show the complete mecular marker in each band. Please consider our situation.

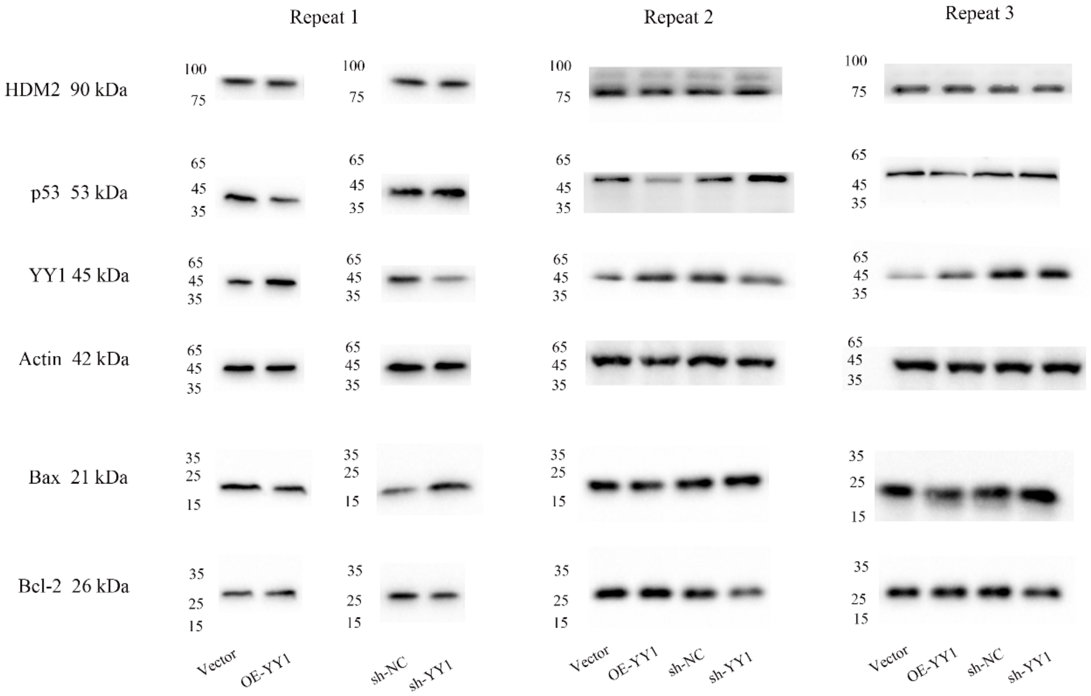

Figure S2 shows the whole blot after cutting membrane at molecular weight 100 kDa, 75 kDa, 65 kDa, 35 kDa and 15 kDa for HDM2 (90 kDa), p53 (53 kDa), YY1 (45 kDa), Actin (42 kDa), Bax (21 kDa) and Bcl-2 (26 kDa). These reprent western blot analysis shown in Fig. 2F.

Since we only added one molecular marker each time in western blot and cut the membrane to many different target bands, we failed to show the complete mecular marker in each band. Please consider our situation.
